# Biological age-acceleration in relation to muscle mass, strength and function: findings from the Hertfordshire Cohort Study

**DOI:** 10.1007/s40520-026-03457-6

**Published:** 2026-07-18

**Authors:** Faidra Laskou, Leo D. Westbury, Negusse Kitaba, John W. Holloway, Elaine M. Dennison, Nicholas R. Fuggle

**Affiliations:** 1 Lifecourse Epidemiology Centre, Faculty of Medicine, University of Southampton, SO16 6YD Southampton, UK; 2 Human Development and Health, Faculty of Medicine, University of Southampton, Southampton, UK

**Keywords:** Age acceleration, Sarcopenia, Grip strength, Gait speed, ALM index

## Abstract

**Introduction:**

Sarcopenia, the accelerated loss of muscle mass, strength and function with age, is associated with adverse health outcomes. DNA methylation accumulates across life and methylation clocks (which provide a measure of biological age) could potentially be used to predict individuals at high sarcopenia risk. In this study, we examined biological age-acceleration measures in relation to sarcopenia components.

**Methods:**

Overall, 331 participants from the UK community-dwelling Hertfordshire Cohort Study were analysed. At baseline (1998–2004), participants underwent health assessments and provided blood samples. At follow-up (2011-12), appendicular lean mass (ALM) index was ascertained using DXA; muscle strength by grip dynamometry; and gait speed over 8ft (2.44 m). DNA was extracted from peripheral blood leukocytes and DNA methylation at CpG sites was measured using the Infinium Human Methylation Beadchip array. Methylation clock measures were ascertained, and examined in relation to grip strength, gait speed and ALM index using linear regression, and in relation to a sarcopenia score (number of sarcopenia components) using Poisson regression. Baseline age, follow-up time, height and BMI were included as adjustments.

**Results:**

At baseline, mean (SD) age was 65.1 (2.8) years. Among males, greater age-acceleration was related to lower grip strength; the standard deviation (SD) difference in grip strength per SD increase in age-acceleration was − 0.16 (-0.32, -0.01) for Principal Component (PC) PhenoAge and − 0.16 (-0.31, -0.01) for bAge. Associations regarding grip strength among females, and for gait speed and ALM index among both males and females were weak. The multiplicative increase in sarcopenia score per SD increase in age-acceleration among females was 1.23 (1.04, 1.45) for PCHorvath1, and 1.20 (1.02, 1.41) for PCHorvath2 (*p* = 0.02 for both associations).

**Conclusions:**

Greater age-acceleration was related to lower grip strength among males only, and associations regarding gait speed and ALM index were weak among both sexes. Greater baseline biological age-acceleration was related to a higher sarcopenia score among females. These findings require further investigation in larger cohorts.

**Supplementary Information:**

The online version contains supplementary material available at https://doi.org/10.1007/s40520-026-03457-6.

## Introduction

Sarcopenia, characterised by the accelerated loss of muscle mass, strength, and function with advancing age, is a prevalent condition among older adults [1]. Sarcopenia is associated with a range of adverse health outcomes, including increased risk of falls, fractures, physical disability, frailty and mortality [2]. The aetiology of sarcopenia is multifactorial (genetic, environmental, and lifestyle factors) [3]. Among the various biological mechanisms implicated in sarcopenia, DNA methylation (cytosine-5 methylation within CpG dinucleotides) has emerged as an area of interest [4].

DNA methylation accumulates progressively throughout an individual’s lifespan [5]. This accumulation can influence gene expression and is associated with age-related diseases [5]. Recent research has introduced the concept of age-acceleration, which refers to the extent to which an individual’s biological age exceeds their chronological age [5]. Age-acceleration is measured using DNA methylation markers and has been proposed as a potential predictor of susceptibility to age-related conditions [6]. The first generation of epigenetic clocks (Horvath 1 [7], Horvath 2 [8], and Hannum [9]) were trained to simply predict chronological age. However, second-generation clocks, such as PhenoAge [10], GrimAge [11], bAge [12] and DNAmFitAge [13], have been developed using bioinformatic approaches which incorporate known lifestyle factors and serum biomarkers of ageing to provide measures of biological age in years and hence can capture both the process of ageing beyond chronological age and provide a measure of biological ageing.

A small number of studies have examined the relationship between some, but not all, sarcopenia components and epigenetic clocks, and different associations were found when different epigenetic clocks were used (Table 1). The Medical Research Council National Survey of Health and Development (NSHD), National Child Development Study (NCDS) and TwinsUK as well as the Lothian Birth Cohort examined associations of age-acceleration and rate of change in DNA methylation with physical function decline [14, 15], while the Health and Retirement Study investigated age acceleration in association with muscle strength decline over 8 years [16]. The Irish Longitudinal Study on Ageing (TILDA), reported age-acceleration in association with walking speed and muscle strength only, while in the MRC National Survey of Health and Development (NSHD) cohort study age-acceleration was measured in relation to muscle strength. Some studies have previously explored the association of several epigenetic clocks with physical functioning (measured by different ways including the Short Physical Performance Battery (SPPB) and five times sit to stand (5-STS)) in older adults (INSPIRE-T and Finnish Twin cohort), showing that GrimAge only was associated with different measures of functional capacity [1, 17, 18].

However, to our knowledge, no studies have examined measures of biological age-acceleration in relation to multiple components of sarcopenia (grip strength, gait speed and appendicular lean mass index) and sarcopenia score, derived from the number of the above components that each participant had. Therefore, we examined this in the Hertfordshire Cohort Study (HCS), a population-based cohort of older males and females [19].

**Table 1:** Summary of the previous clock associations with sarcopenic outcomes

Author	Cohort	Clock measure	Sarcopenic outcome	Effect size	*p*-value
Maddock et al.,[14]	MRC National Survey of Health and Development (NSHD) cohort study & Twins UK	Horvath Hannum PhenoAge GrimAge Horvath Hannum PhenoAge GrimAge	Grip strength Chair rise	-0.02 (-0.25, 0.21) -0.10 (-0.22, 0.02) -0.12 (-0.22, -0.22) -0.03 (-0.14, 0.07) -0.08 (-0.20, 0.05) -0.12 (-0.25, 0.00) -0.20 (-0.41, 0.01) -0.43 (-0.99, 0.14)	0.104 0.496 0.289 0.674 0.61 0.748 0.174 0.068
Marioni et al., [15]	Lothian Birth Cohort	Horvath	Walking speed (6 m) Grip strength- JAMAR	0.03 (0.03) -0.05 (0.02)	0.45 **0.0097**
Peterson et al., [16]	Health and Retirement Study	**Male** PhenoAge GrimAge DunedinPoAm **Female** PhenoAge GrimAge DunedinPoAm	Grip strength (Smedley)	8.27 (14.25, 2.29) 4.56 (8.45, 0.68) 0.36 (0.51, 0.21) 4.96 (2.45, 2.53) 4.46 (7.81, 1.11) 0.36 (0.50, 0.22)	**< 0.05** ** < 0.05** ** < 0.05** > 0.05 ** < 0.05** ** < 0.05**
McCrory et al.,[20]	Irish Longitudinal Study on Ageing (TILDA)	Horvath Hannum PhenoAge GrimAge Horvath Hannum PhenoAge GrimAge	Walking speed (4.88 m) Grip strength (Hydraulic)	-0.62 (-2.37, 1.13) –1.36 (-3.19, 0.48) -1.36 (-3.19, 0.48) -3.74 (-6.03, -1.44) 0.57 (0.07, 1.07) 0.33 (-0.16, 0.83 0.21 (-0.31, 0.72) -0.16 (-0.82, 0.50)	> 0.05 > 0.05 > 0.05 **< 0.001** ** < 0.05** > 0.05 > 0.05 > 0.05
Sanchez et al.,[17]	INSPIRE human translational cohort, Toulouse (INSPIRE T)	Horvath Hannum PhenoAge GrimAge iAge Horvath Hannum PhenoAge GrimAge iAge	Five-­time sit-­to-­stand test Short Physical Performance Battery	−0.006 (− 0.17, 0.16) 0.02 (− 0.14, 0.18) 0.01 (− 0.15, 0.17) 0.25 (0.07, 0.43) 0.16 (− 0.03, 0.34) − 0.02 (− 0.09, 0.06) 0.01 (− 0.06, 0.09) 0.01 (− 0.07, 0.08) − 0.10 (− 0.18, − 0.02) − 0.08 (− 0.16, 0.01)	0.940 0.811 0.929 **0.005** 0.096 0.63 0.70 0.86 **0.019** 0.082
Fohr et al.,[18]	Finnish Twin study on Aging (FITSA)	Horvath Hannum PhenoAge GrimAge Horvath Hannum PhenoAge GrimAge Horvath Hannum PhenoAge GrimAge Horvath Hannum PhenoAge GrimAge	Time up and go (TUG) Walking speed (10 m) Walking speed (6 m) Grip strength	0.000 (0.001) 0.001 (0.001) 0.001(0.001) 0.005 (0.002) 0.000 (0.000) 0.001 (0.000) 0.001 (0.000) 0.001 (0.001) -0.001 (0.001) -0.002 (0.001) -0.001 (0.001) -0.006 (0.002) 0.013 (0.007) 0.000 (0.007) 0.007 (0.005) -0.012 (0.012)	0.777 0.291 0.165 **0.003** 0.882 0.131 **0.126** **0.05** 0.258 0.165 0.082 ** < 0.001** 0.061 0.23 0.184 0.317
Simpkin et al., 2017[21]	NSHD	Horvath blood Horvath buccal	Grip strength Chair rise Grip strength Chair rise	-0.42 (-0.82, 0.02) 0.06 (-0.40, 0.52) -0.02 (-0.16, 012) -0.05 (-0.20, 010)	**0.03** 0.80 0.35 0.53

bAge : Biological Age; standardised, blood and saliva, Principal Component (PC) PhenoAge, PhenoAge: Biological age, based on clinical biomarkers, blood tissue, PCPhenoAge: PhenoAge based on PC component

## Methods

### Hertfordshire Cohort Study

The HCS comprises 2997 participants (53% males) born in Hertfordshire (UK) from 1931 to 1939, who were still living in the county from 1998 to 2004. During this time, participants underwent a comprehensive characterisation of their health which included a home interview and a research clinic visit, to collect data on their sociodemographic, lifestyle, and clinical characteristics. Detailed information about the study has been published previously [19, 22].

**Fig. 1:**
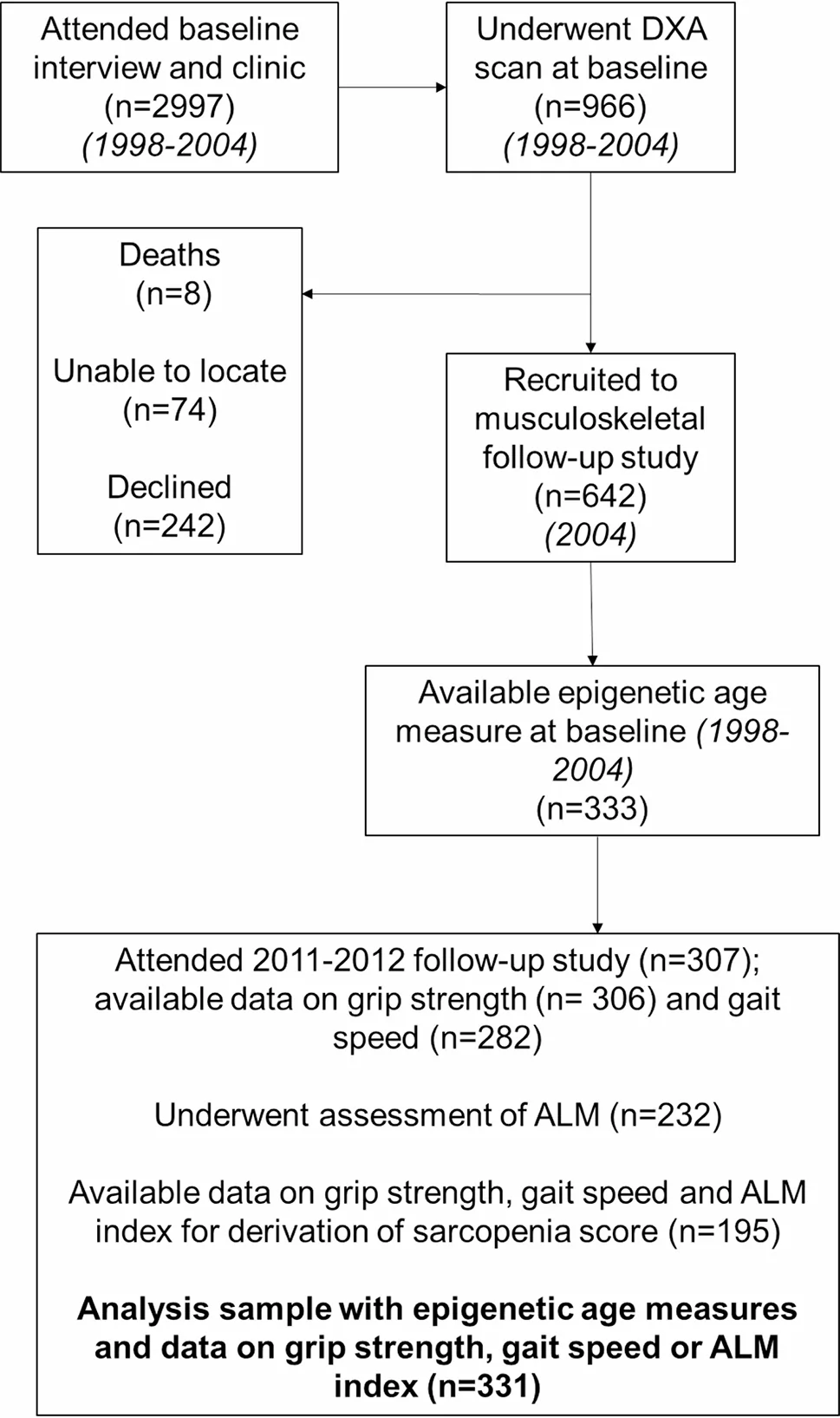
Flow diagram from the Hertforshire Cohort Study baseline stage to the analysis sample. ALM: appendicular lean mass

Of the original cohort, 966 participants from East Hertfordshire had DXA scans at baseline. In 2004, 642/966 were recruited for a musculoskeletal follow-up study. A further follow-up study took place in 2011–2012, inviting 591 participants; 443/591 agreed to participate. During this study, 350/443 underwent detailed body composition assessment, along with an additional 26 HCS participants (*n* = 376 in total). Epigenetic age measures were derived for 333 participants using samples ascertained at HCS baseline (1998–2004). The analysis sample comprised participants with epigenetic age measures who also had values for grip strength, gait speed or appendicular lean mass index. A flow diagram from the HCS baseline cohort (1998–2004) to the analysis sample is presented in Fig. 1.

### Ascertainment of participant information at baseline (1998–2004)

Information on alcohol consumption, smoking status and physical activity (Dallosso questionnaire [23] was collected at the home interview through a clinician-administered questionnaire. Diet was assessed using a food frequency questionnaire (FFQ), administered by a trained research nurse. The foods in the FFQ were combined into 51 food groups on the basis of similarity of type of food and nutrient composition. A ‘prudent’ diet score was calculated by applying principal component analysis to reported weekly frequencies of consumption of these food groups; healthier diets were indicated by higher diet scores [24].

At the baseline clinic, height was measured (Harpenden pocket stadiometer, Chasmors Ltd, London, UK) along with weight (SECA floor scale, Chasmors Ltd, London, UK) and used to derive body mass index (BMI). Handgrip strength was assessed three times on each side using a Jamar dynamometer; the highest of the six measurements was used for analysis. Blood samples were taken and serum was aliquoted and stored at -80 °C.

### Ascertainment of participant information at follow-up (2011–2012)

Height and handgrip strength were measured using the same equipment and protocol as at baseline. Mean customary walking speed in meters per second was ascertained using the mean time from two 8ft (2.44 m) gait speed tests. Appendicular lean mass (ALM) was derived using DXA (Lunar Prodigy Advanced Scanner, GE Medical Systems, UK); this measure was used, along with the square of height (m) at this time-point, to derive appendicular lean mass index (ALMi) in kg/m^2^. A sarcopenia score (0–3) was also derived from the number of the following components that each participant had: low grip strength (males < 27 kg, females < 16 kg); low gait speed (≤ 0.8 m/s); and low ALMi (males < 7.0 kg/m^2^, females < 5.5 kg/m^2^). These thresholds were based on the 2019 European Working Group on Sarcopenia in Older People (EWGSOP2) definition [1].

### Derivation of epigenetic age-acceleration measures

DNA methylation was measured in DNA extracted from peripheral blood leukocytes using a simple salting-out procedure [25]. Bisulfite-conversion was undertaken using EZ 96-DNA methylation kits (Zymo Research, Irvine, CA, USA). Methylation was assessed using Illumina Infinium Methylation EPIC Beadchip arrays (Illumina, Inc. CA, USA) with samples randomly distributed on microarrays to control for batch effects. Methylation intensity files were processed, and quality was assessed using minfi [26] in R (version 4.4.1). Probe normalization and filters were conducted using ref-RCP in the Enmix package [27]. Epigenetic predictor scores were generated using the methscore function [28] from the ENmix package using the methylation beta values as an input.

Calculating the difference between each epigenetic age measure and a participant’s chronological age at the HCS baseline clinic (1998–2004) was used to derive residual variables for each epigenetic age measure. These residual variables are measures of epigenetic age-acceleration; positive values (above zero) for a particular residual measure would indicate that a participant’s biological age is greater (older) than their chronological age, negative values (below zero) would indicate that a participant’s biological age is less (younger) than their chronological age.

### Statistical methods

Summary statistics were used to describe the participant characteristics at the HCS baseline (1998–2004) and follow-up stage (2011–2012). Each baseline epigenetic age-acceleration measure was examined in relation to grip strength, gait speed and ALMi using sex-stratified linear regression; Poisson regression with robust variance estimation was used for the sarcopenia score outcome. Advantages of this technique are that it yields interpretable effect estimates (multiplicative increases in the outcome according to the exposure) and relaxes the strict Poisson distribution assumption that the mean and the variance of the outcome are equal. Follow-up time and baseline age, height and BMI were included as adjustments; sensitivity analyses additionally included smoking status, alcohol consumption, physical activity and diet quality as adjustments. Further sensitivity analyses included examining the grip strength outcome at follow-up after adjustment for follow-up time and baseline grip strength, age, height and BMI. Associations were also interpreted after Bonferroni correction, revising the standard significance level threshold of 0.05 to 0.00057, given the 88 statistical tests performed (examination of 11 epigenetic age exposures in relation to 4 outcomes among men and women separately). All analyses were performed using Stata (release 17.0). Effect sizes are reported along with 95% confidence intervals. Post hoc adjustment for leukocyte fractions would remove meaningful variance so we have not adjusted for cell types.

## Results

### Participant characteristics at baseline (1998–2004) and follow-up (2011–2012)

Participants' characteristics are presented in Table 2. Mean (SD) age at baseline among males and females was 64.2 (2.6) and 65.9 (2.8) respectively. At follow-up, all outcomes were higher among males compared to females: grip strength (36.1 (7.4) kg versus 20.8 (5.9)), gait speed (0.79 (0.18) m/s versus 0.73 (0.19)) and ALMi (8.1 (0.7) kg/m^2^ versus 6.4 (0.7)).

**Table 2:** Participant characteristics of the analysis sample

Participant characteristic	Mean (SD); median (lower quartile, upper quartile) or *n* (%)		Missing values
	Males (*n* = 165)	Females (*n* = 166)	
***HCS Baseline (1998–2004)***			
Age (years)	64.2 (2.6)	65.9 (2.8)	0
Height (cm)	174.3 (6.6)	161.3 (6.1)	0
Weight (kg)	81.3 (11.3)	70.0 (13.2)	0
BMI (kg/m^2^)	26.8 (3.6)	26.9 (4.7)	0
Ever smoked	91 (55%)	60 (36%)	0
Alcohol consumption (units per week)	10.0 (2.6, 20.5)	1.5 (0.0, 4.5)	0
Dallosso physical activity score	63.8 (14.5)	61.6 (13.4)	0
Prudent diet score	-0.6 (2.0)	1.0 (1.7)	0
Grip strength	44.9 (7.0)	27.4 (4.8)	0
***HCS follow-up (2011–2012)***			
Grip strength (kg)	36.1 (7.4)	20.8 (5.9)	25
Gait speed (m/s)	0.79 (0.18)	0.73 (0.19)	49
Appendicular lean mass index (kg/m^2^)	8.1 (0.7)	6.4 (0.7)	99
Sarcopenia score*			136
0	51 (51.0%)	29 (30.5%)	
1	44 (44.0%)	50 (52.6%)	
2	5 (5.0%)	15 (15.8%)	
3	0 (0.0%)	1 (1.1%)	

Sarcopenia score: number of the following: low grip strength (males < 27 kg, females < 16 kg); low gait speed (≤ 0.8 m/s); and low appendicular lean mass index (males < 7.0 kg/m^2^, females < 5.5 kg/m^2^)

Supplementary Table 1 compares the participant characteristics at HCS baseline (1998–2004) between those who were included in the analysis sample and those who were not. The following differences (*p* < 0.05) were observed for those who were included compared to those who were not: younger age, lower BMI (females only), lower prevalence of ever smokers (males only), higher physical activity, higher diet quality (females only), and higher grip strength (females only).

### Epigenetic age-acceleration measures in relation to outcomes (grip strength, gait speed, ALMi, sarcopenia score)

These associations are presented in Table 3. Among males, greater age-acceleration according to measures PCPhenoAge and bAge was related to lower grip strength after adjustment for follow-up time and baseline age, height and BMI. For example, the standard deviation (SD) difference in grip strength per SD increase in age-acceleration was − 0.16 (-0.32, -0.01) for PCPhenoAge, and − 0.16 (-0.31, -0.01) for bAge. Among females only, greater age-acceleration according to PCHorvath1 and PCHorvath2 was related to higher values for the sarcopenia score. For example, the multiplicative increase in sarcopenia score per SD increase in age-acceleration among females was 1.23 (1.04, 1.45) for PCHorvath1, and 1.20 (1.02, 1.41) for PCHorvath2.

Supplementary Table 2 presents the associations between the epigenetic age-acceleration measures and outcomes when the initial set of adjustments (follow-up time and baseline age, height and BMI) was extended to include baseline smoking status, alcohol consumption, physical activity and diet quality. None of these associations were statistically significant for the outcome variables grip strength, gait speed and ALMi. However, associations between PCHorvath1 and PCHorvath2 and sarcopenia score among females remained robust in these sensitivity analyses.

Supplementary Table 3 presents associations for the grip strength outcome at follow-up after additional adjustment for baseline grip strength. The previous associations detailed above for PCPhenoAge and bAge in relation to grip strength at follow-up among men were weak when adjusted for baseline grip strength (*p* > 0.38 for both associations).

After implementing Bonferroni correction, none of the associations examined were statistically significant (*p* > 0.00057 for all associations).

**Table 3:** SD difference in outcome at follow-up per SD increase in baseline age-acceleration exposure (stratified by sex)

Exposure	Grip strength (z-score)		Gait speed (z-score)		ALMi (z-score)		Sarcopenia score	
	Estimate (95% CI)	*P*-value	Estimate (95% CI)	*P*-value	Estimate (95% CI)	*P*-value	Estimate (95% CI)	*P*-value
***Males***								
HorvathAge	0.05 (-0.11,0.20)	0.56	0.08 (-0.08,0.24)	0.31	-0.06 (-0.20,0.08)	0.43	1.00 (0.81,1.23)	0.97
Horvath2	0.02 (-0.13,0.18)	0.79	0.04 (-0.12,0.20)	0.62	0.08 (-0.07,0.22)	0.31	1.05 (0.87,1.28)	0.61
HannumAge	-0.02 (-0.18,0.13)	0.75	0.06 (-0.09,0.22)	0.43	0.09 (-0.06,0.23)	0.23	1.10 (0.90,1.34)	0.35
PCHorvath1	-0.04 (-0.20,0.12)	0.63	0.11 (-0.05,0.27)	0.17	0.12 (-0.03,0.26)	0.12	1.05 (0.86,1.28)	0.62
PCHorvath2	-0.01 (-0.16,0.15)	0.94	0.11 (-0.05,0.27)	0.16	0.14 (-0.00,0.29)	0.06	0.98 (0.80,1.21)	0.87
PCHannum	-0.04 (-0.20,0.11)	0.57	0.11 (-0.05,0.26)	0.18	0.11 (-0.03,0.26)	0.13	1.00 (0.83,1.20)	0.96
PhenoAge	-0.11 (-0.26,0.03)	0.13	0.02 (-0.14,0.17)	0.85	-0.06 (-0.22,0.09)	0.41	1.10 (0.91,1.34)	0.31
PCPhenoAge	**-0.16 (-0.32**,**-0.01)**	**0.04**	0.04 (-0.12,0.20)	0.65	0.03 (-0.12,0.19)	0.68	1.10 (0.91,1.35)	0.32
PCGrimAge	-0.12 (-0.28,0.03)	0.12	0.01 (-0.15,0.18)	0.86	-0.03 (-0.17,0.12)	0.70	1.01 (0.83,1.22)	0.91
bAge	**-0.16 (-0.31**,**-0.01)**	**0.04**	0.00 (-0.16,0.17)	0.98	-0.01 (-0.16,0.13)	0.85	1.09 (0.92,1.29)	0.34
DNAmFitAge	-0.11 (-0.26,0.04)	0.16	0.11 (-0.05,0.27)	0.18	0.00 (-0.15,0.15)	0.98	0.92 (0.76,1.13)	0.43
***Females***								
HorvathAge	0.08 (-0.08,0.24)	0.34	0.04 (-0.12,0.21)	0.60	0.01 (-0.14,0.15)	0.94	1.05 (0.90,1.24)	0.54
Horvath2	-0.01 (-0.17,0.15)	0.88	-0.03 (-0.20,0.13)	0.69	-0.05 (-0.20,0.10)	0.51	1.15 (0.98,1.34)	0.08
HannumAge	0.12 (-0.04,0.28)	0.15	0.07 (-0.10,0.23)	0.43	0.02 (-0.13,0.16)	0.81	1.09 (0.90,1.32)	0.37
PCHorvath1	-0.00 (-0.16,0.16)	0.97	-0.05 (-0.21,0.11)	0.54	0.02 (-0.13,0.16)	0.81	**1.23 (1.04**,**1.45)**	**0.02**
PCHorvath2	-0.00 (-0.16,0.16)	0.98	-0.02 (-0.18,0.14)	0.78	0.05 (-0.09,0.19)	0.49	**1.20 (1.02**,**1.41)**	**0.02**
PCHannum	0.07 (-0.09,0.23)	0.40	0.07 (-0.09,0.24)	0.38	0.05 (-0.09,0.19)	0.46	1.12 (0.94,1.34)	0.20
PhenoAge	0.05 (-0.12,0.21)	0.58	0.01 (-0.15,0.17)	0.93	-0.04 (-0.18,0.10)	0.56	1.08 (0.91,1.29)	0.36
PCPhenoAge	0.05 (-0.10,0.21)	0.49	-0.07 (-0.23,0.09)	0.37	0.03 (-0.11,0.16)	0.70	1.11 (0.94,1.31)	0.23
PCGrimAge	0.07 (-0.09,0.23)	0.42	-0.06 (-0.22,0.10)	0.47	0.07 (-0.06,0.21)	0.27	1.00 (0.84,1.21)	0.96
bAge	0.08 (-0.08,0.24)	0.31	-0.05 (-0.20,0.11)	0.57	0.08 (-0.06,0.21)	0.25	1.00 (0.83,1.20)	0.96
DNAmFitAge	0.04 (-0.12,0.20)	0.61	-0.05 (-0.21,0.11)	0.57	0.01 (-0.14,0.15)	0.92	1.07 (0.89,1.29)	0.49

Sarcopenia score: number of the following: low grip strength (males < 27 kg, females < 16 kg); low gait speed (≤ 0.8 m/s); and low appendicular lean mass index (males < 7.0 kg/m^2^, females < 5.5 kg/m^2^). For this outcome, estimates correspond to the multiplicative increase in the outcome per SD increase in the exposure; an estimate of 1.2 corresponds to a 20% increase in the outcome and an estimate of 0.8 corresponds to a 20% decrease

SD Standard deviation; CI Confidence interval; ALMi Appendicular lean mass index

All models were adjusted for follow-up time and baseline age, height and BMI

Positive estimates indicate that greater age-acceleration according to the corresponding measure was related to higher values of outcomes; negative estimates indicate that these were related to lower values of outcomes

Number of observations for outcomes were as follows: grip strength (men *n* = 151, women *n* = 155), gait speed (men *n* = 140, women *n* = 142), ALMi (men *n* = 119, women *n* = 113), sarcopenia score (men *n* = 100, women *n* = 95)

## Discussion

In this population-based cohort of community-dwelling older people, we observed that greater baseline biological age-acceleration (older biological than chronological age) according to PCPhenoAge and bAge was related to lower grip strength among males assessed around one decade later after adjustment for follow-up time, chronological age, height and BMI. However, associations for grip strength among females, and for gait speed and ALMi among both males and females were weak. Furthermore, none of the associations for grip strength, gait speed and ALMi were robust after additional adjustment for baseline smoking status, alcohol consumption, physical activity and diet quality. However, greater baseline biological age-acceleration according to PCHorvath1 and PCHorvath2 was related to a higher sarcopenia score among females; these associations were robust regardless of which set of adjustments was used.

Our study is the first to examine all sarcopenic components and the sarcopenia score, reflecting severity of sarcopenia, in relation to age-acceleration. In comparison to previous studies, we included muscle strength, muscle quantity and physical performance in association with epigenetic clocks. We have also examined these associations with all available epigenetic clocks (first and second generation) in both males and females making it possible to assess sex-specific associations.

Neither the age-acceleration at baseline nor the rate of change in DNA methylation, using Horvath’s first generation clock, predicted physical function decline, measured by handgrip strength, chair rise or chair-stand test over 6 and 16 years in the Medical Research Council National Survey of Health and Development (NSHD), the National Child Development Study (NCDS), and TwinsUK as well as in the Lothian Birth Cohort [14, 15]. However, Hannum’s clock at baseline was associated with reduced handgrip strength in the Lothian Birth Cohort, while PhenoAge and GrimAge were associated with slower chair rise time; PhenoAge alone was associated with handgrip strength decline at 16 years follow-up [14]. Sex-specific associations were not reported in these studies.

In the Health and Retirement Study, accelerated ageing, measured by PhenoAge and GrimAge, predicted decline in muscle strength at 8 years follow up; however, this was observed only in males [16], as aligned with our findings.

Diverging from our findings, associations between grip strength and acceleration clocks were not consistent across sex categories in fully-adjusted models in the Health and Retirement study [16]. Similarly, in the Irish Longitudinal Study on Ageing (TILDA), higher GrimAge acceleration was associated with slower walking speed but not with decline in grip strength [20]. In an analysis restricted to females (the MRC National Survey of Health and Development (NSHD) cohort study), age-acceleration, measured using the Horvarth method, was associated with a greater decrease in grip strength [21].

Some studies have previously explored the association of epigenetic clocks, with physical functioning (measured by different ways including SPPB and 5-STS) in older adults (INSPIRE-T and Finnish Twin cohort); worse SPPB was associated with PhenoAge and GrimAge in males older than 84, in males older than 74, and among females in the age range 58–86, respectively, in the INSPIRE T study [17], while in the NSHD cohort study, where only females were examined, no association was found between age acceleration and standing balance or chair rise speed [21].

Studies have linked the increase in epigenetic clocks with worse handgrip strength [15, 21, 30], while others point to the absence of significant associations with the latter and focus on physical capacity-related endpoints [14, 17, 18, 30]. Previous observational research suggests an accelerated biological ageing rate among males compared to females [31], while females may carry a higher disability burden and frailty severity [32, 33]. Males, compared to females, are found to have higher innate and pro-inflammatory activity and lower adaptive activity; a pattern that is thought to contribute to Inflammaging [34]. Such divergences may reflect differences in biological phenotypes, genetic foundations, and physiological characteristics between males and females [35]. First generation clocks primarily capture chronological age-associated changes; their association with sarcopenia severity in females but not males may stem from key biological and hormonal differences. Oestrogen exerts anti-inflammatory effects and preserves muscle mass; during menopause, rapid oestrogen decline triggers systemic inflammation and accelerated muscle catabolism. In females, this translates to a stronger correlation between chronological based methylation shifts and decline in muscle mass [36]. Studies have shown differences in mitochondria size, intramuscular carnitine, myostatin, and IGF1 levels between males and females suggesting a variety of biological processes associated with sarcopenia and ageing [4, 37]. It is though important to consider, that some of the sex associated differences observed in our study could be due to chance findings especially given the available small sample sizes when stratified by sex.

It is important to note that differences between our findings and those from previous studies may be due to variations in the methodological approaches used. For example, differences in sarcopenia definitions, epigenetic clock derivation procedures, and in adjustments included in statistical models may contribute to differences in the findings observed between studies.

This study has several strengths. A broad set of epigenetic age-acceleration measures and sarcopenia components were considered in this study. In addition, the analysis accounted for a broad set of participant characteristics which were included as adjustments in regression models. Furthermore, the HCS has rigorous phenotyping protocols and was conducted by well-trained fieldworkers and managed by an experienced multidisciplinary team. However, this study does have some limitations. For instance, a healthy participant effect is evident in the HCS cohort [22] and attrition across follow-up stages could have resulted in further selection effects and bias. For example, participants who were included in the analysis sample were younger at baseline (1998–2004) and generally had better health behaviours at baseline compared to those who were not included (Supplementary Table 1). Nevertheless, the baseline cohort was found to be broadly comparable to participants in the nationally representative Health Survey for England [22]. Second, the study’s sample size was relatively small, though associations between greater biological age-acceleration and lower grip strength among males have been reported in previous studies. This reduces the statistical power to detect associations. For example, in a simple Poisson regression model for the sarcopenia score among the males, a large minimum detectable effect of a 36% increase in the sarcopenia score per standard deviation increase in the predictor would be required to achieve 80% power at the 5% significance level. A limitation is that participants were included based on available grip strength, gait speed or ALMi data, resulting in differing levels of missing data across outcomes, with the largest proportion missing for ALMi index and sarcopenia score. Another potential limitation is the fact that epigenetic clocks were not muscle-specific in this study, taking into account that age-related conditions are often associated with tissue‐specific effects [38]. The potential for reverse causation also cannot be excluded; indeed, associations between greater age-acceleration according to PCPhenoAge and bAge and lower grip strength at follow-up among men were weak when adjusted for baseline grip strength. Another limitation is that gait speed was only assessed over 8ft (2.44 m); this differs from distances used in previous studies and those proposed by EWGSOP2 [1], potentially limiting the comparability of our findings with the wider literature. Finally, a potential limitation is the use of multiple clocks (*n* = 11), which may introduce a multiple-testing burden and increase the risk of false positives. For example, the application of Bonferroni correction attenuated all associations to non-significance. Accordingly, these findings should be interpreted as exploratory, and not as definitive evidence of associations between specific epigenetic clocks and components of sarcopenia. However, the implementation of Bonferroni correction may be regarded as overly stringent in this context, given the correlation between the epigenetic clocks.

## Conclusion

In this cohort of community-dwelling older adults, greater biological age-acceleration, particularly as measured by PCPhenoAge and bAge, was associated with lower grip strength among males over a decade of follow-up, while grip strength associations for females, and for other sarcopenia components (gait speed and appendicular lean mass index) were weak. Our study is the first to comprehensively examine multiple sarcopenia components alongside a wide range of epigenetic clocks in both sexes, highlighting potential sex-specific differences. Our study was also the first to examine age acceleration in relation to sarcopenia severity measured by the sarcopenia score indicating that greater baseline biological age-acceleration was related to greater sarcopenia severity among females. Larger, more diverse cohorts and mechanistic studies are needed to clarify the role of epigenetic ageing in sarcopenia development and progression, and to confirm the described sex specific associations.

## Supplementary Information

Below is the link to the electronic supplementary material.

Supplementary Material 1

## Data Availability

The data used in the current study cannot be shared due to consent restrictions..
