# Supplementary material for: Biological age-acceleration in relation to muscle mass, strength and function: findings from the Hertfordshire Cohort Study
Source: Aging Clin Exp Res. 2026 Jul 18;38(1):214. doi: 10.1007/s40520-026-03457-6 (PMC13672666; doi:10.1007/s40520-026-03457-6)
Supplement: Supplementary file 1 [file 40520_2026_3457_MOESM1_ESM.docx]

**Biological age-acceleration in relation to muscle mass, strength and function: findings from the Hertfordshire Cohort Study**

Faidra Laskou^1^, Leo D Westbury^1^, Negusse Kitaba^2^, John W Holloway^2^, Elaine M Dennison^1^, Nicholas R Fuggle^1^

**Affiliations**

*^1^MRC Lifecourse Epidemiology Centre, University of Southampton, Southampton, UK*

*^2^Human Development and Health, Faculty of Medicine, University of Southampton, Southampton, UK*

**Correspondence**

**Faidra Laskou ;** [f.laskou@s**oton.ac.uk**](mailto:f.laskou@soton.ac.uk)

**MRC Lifecourse Epidemiology Centre, University of Southampton , Southampton, UK**

Supplementary tables

| **Supplementary Table 1: Comparison of participant characteristics at baseline (1998-2004) between the analysis sample and the group of participants who were not included in the analysis sample, stratified by sex** | | | | | |
| --- | --- | --- | --- | --- | --- |
| **Participant characteristic at baseline (1998-2004)** | **Mean (SD); median (lower quartile, upper quartile); or n(%)** | | | |  |
|  | **Males** | | **Females** | |  |
|  | **Group included in analysis sample (n=165)** | **Group not included in analysis sample (n=1414)** | **Group included in analysis sample (n=166)** | **Group not included in analysis sample (n=1252)** |  |
| Age (years)^a b^ | 64.2 (2.6) | 65.9 (2.9) | 65.9 (2.8) | 66.7 (2.7) |  |
| BMI (kg/m^2^) ^b^ | 26.8 (3.6) | 27.2 (3.8) | 26.9 (4.7) | 27.7 (4.9) |  |
| Ever smoked ^a^ | 91 (55%) | 968 (68%) | 60 (36%) | 493 (39%) |  |
| Alcohol intake (units per week) | 10.0 (2.6, 20.5) | 8.5 (2.0, 18.8) | 1.5 (0.0, 4.5) | 1.5 (0.0, 6.0) |  |
| Dallosso physical activity score ^a b^ | 63.8 (14.5) | 60.6 (15.4) | 61.6 (13.4) | 58.6 (16.0) |  |
| Prudent diet score ^b^ | -0.6 (2.0) | -0.6 (2.1) | 1.0 (1.7) | 0.7 (1.7) |  |
| Grip strength (kg) ^b^ | 44.9 (7.0) | 43.9 (7.6) | 27.4 (4.8) | 26.4 (5.9) |  |
| *^a^ Difference between groups (p<0.05) among men*  *^b^ Difference between groups (p<0.05) among women* | | | | | |

**Supplementary Table 2: SD difference in outcome at follow-up per SD increase in baseline age-acceleration exposure (stratified by sex)**

| **Exposure** | **Grip strength (z-score)** | | **Gait speed (z-score)** | | **ALMi (z-score)** | | | **Sarcopenia score** | |
| --- | --- | --- | --- | --- | --- | --- | --- | --- | --- |
|  | **Estimate (95% CI)** | **P-value** | **Estimate (95% CI)** | **P-value** | | **Estimate (95% CI)** | **P-value** | **Estimate (95% CI)** | **P-value** |
| ***Males*** |  |  |  |  | |  |  |  |  |
| HorvathAge | 0.04 (-0.11,0.20) | 0.59 | 0.07 (-0.09,0.23) | 0.41 | | -0.06 (-0.19,0.08) | 0.44 | 1.03 (0.83,1.29) | 0.78 |
| Horvath2 | 0.06 (-0.10,0.22) | 0.45 | 0.05 (-0.10,0.21) | 0.50 | | 0.09 (-0.05,0.24) | 0.20 | 1.04 (0.87,1.24) | 0.65 |
| HannumAge | -0.03 (-0.18,0.13) | 0.74 | 0.07 (-0.09,0.23) | 0.38 | | 0.08 (-0.06,0.22) | 0.28 | 1.06 (0.87,1.29) | 0.55 |
| PCHorvath1 | -0.03 (-0.19,0.13) | 0.73 | 0.10 (-0.06,0.26) | 0.21 | | 0.10 (-0.05,0.24) | 0.19 | 1.02 (0.84,1.23) | 0.87 |
| PCHorvath2 | -0.01 (-0.16,0.15) | 0.93 | 0.11 (-0.05,0.27) | 0.18 | | 0.12 (-0.02,0.27) | 0.10 | 0.93 (0.76,1.14) | 0.47 |
| PCHannum | -0.04 (-0.19,0.12) | 0.65 | 0.10 (-0.06,0.25) | 0.22 | | 0.10 (-0.04,0.24) | 0.17 | 0.96 (0.80,1.16) | 0.70 |
| PhenoAge | -0.10 (-0.25,0.05) | 0.21 | 0.00 (-0.15,0.16) | 0.98 | | -0.08 (-0.23,0.07) | 0.31 | 1.11 (0.90,1.36) | 0.32 |
| PCPhenoAge | -0.14 (-0.30,0.01) | 0.06 | 0.03 (-0.13,0.19) | 0.68 | | 0.02 (-0.14,0.17) | 0.84 | 1.06 (0.86,1.31) | 0.56 |
| PCGrimAge | -0.09 (-0.27,0.09) | 0.32 | 0.04 (-0.14,0.23) | 0.67 | | -0.09 (-0.25,0.08) | 0.31 | 0.95 (0.77,1.18) | 0.66 |
| bAge_Years | -0.13 (-0.31,0.04) | 0.13 | 0.03 (-0.16,0.21) | 0.77 | | -0.06 (-0.23,0.11) | 0.49 | 1.02 (0.83,1.25) | 0.84 |
| DNAmFitAge | -0.08 (-0.24,0.08) | 0.32 | 0.12 (-0.05,0.28) | 0.16 | | -0.00 (-0.15,0.15) | 0.99 | 0.90 (0.73,1.10) | 0.29 |
|  |  |  |  |  | |  |  |  |  |
| ***Females*** |  |  |  |  | |  |  |  |  |
| HorvathAge | 0.06 (-0.10,0.22) | 0.47 | 0.01 (-0.15,0.17) | 0.91 | | -0.00 (-0.15,0.15) | 0.99 | 1.08 (0.93,1.25) | 0.33 |
| Horvath2 | -0.03 (-0.19,0.12) | 0.68 | -0.05 (-0.21,0.11) | 0.50 | | -0.06 (-0.21,0.09) | 0.45 | **1.17 (1.01,1.35)** | **0.04** |
| HannumAge | 0.11 (-0.04,0.27) | 0.15 | 0.06 (-0.10,0.22) | 0.44 | | 0.01 (-0.13,0.16) | 0.85 | 1.07 (0.92,1.25) | 0.37 |
| PCHorvath1 | 0.01 (-0.15,0.17) | 0.91 | -0.05 (-0.20,0.11) | 0.56 | | 0.02 (-0.13,0.16) | 0.80 | **1.21 (1.05,1.39)** | **0.01** |
| PCHorvath2 | 0.02 (-0.14,0.17) | 0.83 | -0.00 (-0.16,0.15) | 0.96 | | 0.06 (-0.08,0.20) | 0.42 | **1.16 (1.02,1.33)** | **0.02** |
| PCHannum | 0.07 (-0.09,0.22) | 0.41 | 0.09 (-0.07,0.25) | 0.28 | | 0.05 (-0.09,0.20) | 0.45 | 1.11 (0.96,1.28) | 0.15 |
| PhenoAge | 0.06 (-0.10,0.21) | 0.47 | 0.03 (-0.13,0.18) | 0.74 | | -0.03 (-0.17,0.11) | 0.69 | 1.07 (0.93,1.23) | 0.33 |
| PCPhenoAge | 0.07 (-0.08,0.22) | 0.39 | -0.03 (-0.19,0.13) | 0.69 | | 0.02 (-0.12,0.16) | 0.76 | 1.10 (0.95,1.28) | 0.21 |
| PCGrimAge | 0.13 (-0.03,0.29) | 0.11 | 0.02 (-0.14,0.19) | 0.80 | | 0.05 (-0.09,0.20) | 0.45 | 1.00 (0.84,1.19) | 0.97 |
| bAge_Years | 0.13 (-0.03,0.28) | 0.11 | 0.02 (-0.14,0.18) | 0.79 | | 0.06 (-0.08,0.20) | 0.41 | 1.00 (0.84,1.18) | 0.96 |
| DNAmFitAge | 0.06 (-0.10,0.22) | 0.45 | -0.01 (-0.17,0.15) | 0.87 | | -0.01 (-0.16,0.14) | 0.89 | 1.06 (0.91,1.24) | 0.45 |
| *Sarcopenia score: number of the following: low grip strength (males <27 kg, females <16 kg); low gait speed (≤0.8 m/s); and low appendicular lean mass index (males <7.0 kg/m^2^, females <5.5 kg/m^2^). For this outcome, estimates correspond to the multiplicative increase in the outcome per SD increase in the exposure; an estimate of 1.2 corresponds to a 20% increase in the outcome and an estimate of 0.8 corresponds to a 20% decrease.* | | | | | | | | | |
| *SD Standard deviation; CI Confidence interval; ALMi Appendicular lean mass index* | | | | | | | | | |
| *All models were adjusted for follow-up time and the following baseline characteristics: age, height, BMI, smoking status, alcohol consumption, physical activity and diet quality* | | | | | | | | | |
|  | | | | | | | | | |

| **Supplementary Table 3: Standard deviation difference in grip strength at follow-up per standard deviation increase in baseline age-acceleration exposure after adjustment for follow-up time and baseline grip strength, age, height and BMI** | | | | |
| --- | --- | --- | --- | --- |
| **Exposure** | **Males** | | **Females** | |
|  | **Estimate (95% CI)** | **P-value** | **Estimate (95% CI)** | **P-value** |
| HorvathAge | 0.04 (-0.08,0.16) | 0.48 | 0.05 (-0.08,0.18) | 0.43 |
| Horvath2 | -0.02 (-0.14,0.10) | 0.79 | 0.01 (-0.12,0.14) | 0.88 |
| HannumAge | -0.06 (-0.18,0.06) | 0.31 | 0.13 (0.01,0.26) | 0.04 |
| PCHorvath1 | 0.00 (-0.12,0.13) | 0.94 | 0.04 (-0.09,0.17) | 0.56 |
| PCHorvath2 | 0.03 (-0.09,0.15) | 0.61 | 0.04 (-0.09,0.17) | 0.54 |
| PCHannum | 0.00 (-0.12,0.12) | 0.96 | 0.09 (-0.04,0.22) | 0.18 |
| PhenoAge | -0.04 (-0.16,0.07) | 0.48 | 0.07 (-0.06,0.20) | 0.28 |
| PCPhenoAge | -0.05 (-0.17,0.07) | 0.39 | 0.10 (-0.03,0.22) | 0.12 |
| PCGrimAge | -0.03 (-0.15,0.09) | 0.62 | 0.06 (-0.07,0.19) | 0.34 |
| bAge_Years | -0.03 (-0.16,0.09) | 0.58 | 0.09 (-0.04,0.21) | 0.17 |
| DNAmFitAge | -0.04 (-0.16,0.07) | 0.46 | 0.05 (-0.08,0.18) | 0.47 |
| *Positive estimates indicate that greater age-acceleration according to corresponding measure was related to higher grip strength at follow-up; negative estimates indicate that these were related to lower grip strength at follow-up* | | | | |
